# Evaluation of salivary biomarker interleukin-6 in oral squamous cell carcinoma and its clinical correlation

**DOI:** 10.6026/973206300211497

**Published:** 2025-06-30

**Authors:** Dipak Chaudhari, Shilpa Motghare, Vikas Pandey, Gunmeek Kaur, Sameer Gupta, Saptadisha Rakshit, Miral Mehta, Bhumika J. Patel

**Affiliations:** 1 Department of General Dentistry, Smilebuilderz LLC, Lancaster, Pennsylvania, US; 2 Department of Pathology, SRVS, Government Medical College, Shivpuri (Madhya Pradesh), India; 3 Departmnet of Pathology, Goverment Medical College, Satna, Madhya Pradesh, India; 4 Department of Oral and Maxillofacial Surgery, Luxmi Bai Dental College and Hospital, Patiala, Punjab, India; 5 Department of Oral & Maxillofacial Surgery, Inderprastha Dental College and Hospital, Sahibabad , Ghaziabad , Uttar Pradesh - 201010, Ghaziabad, India; 6 Intern batch BDS 2020, Kalinga Institute of Dental Sciences, KIIT Deemed to be University, Odisha, India; 7 Department of Pediatric and Preventive Dentistry, Karnavati School of Dentistry, Karnavati University, Gandhinagar, Gujarat,India; 8 Department of Dentistry, Howard University, College of Dentistry, Washington DC, USA

**Keywords:** Oral squamous cell carcinoma, interleukin-6, salivary biomarkers, non-invasive diagnostics, cancer staging

## Abstract

Oral Squamous Cell Carcinoma (OSCC) is a prevalent malignancy with high morbidity. Hence, this cross-sectional study evaluated salivary interleukin-6 (IL-6) levels in 30 OSCC patients versus 30 healthy controls. IL-6 levels were significantly higher in OSCC patients and increased progressively with advancing clinical stages. A strong positive correlation was found between IL-6 levels and disease stage (r = 0.82, p < 0.001). Salivary IL-6 shows promise as a non-invasive biomarker for OSCC detection and progression monitoring.

## Background:

The most prevalent cancer form in the oral cavity is Oral Squamous Cell Carcinoma which represents more than nine out of ten oral cancer cases [1]. The current 5-year survival statistics of Oral Squamous Cell Carcinoma continue to remain low mainly because patients often receive diagnoses too late in the progression of their disease [2]. Although biopsy and imaging work well for diagnosis they remain both invasive and expensive while their repeated use is limited [3]. The diagnostic molecules present within saliva make this readily accessible biofluid an emerging non-invasive medium for early cancer detection because of its DNA RNA protein and cytokine content [4]. Research has thoroughly studied the pro-inflammatory cytokine interleukin-6 (IL-6) for its functions in cancer tumorigenesis and its ability to trigger cell proliferation and angiogenesis along with metastatic processes [5]. Salivary MMP-9 levels are significantly elevated in patients with oral squamous cell carcinoma (OSCC) and oral potentially malignant disorders (OPMD) compared to tobacco users and healthy individuals. MMP-9 concentrations correlate with the degree of tumor differentiation, suggesting a role in assessing disease severity and progression. Salivary MMP-9 shows high sensitivity and moderate specificity, indicating its potential as a non-invasive biomarker for early diagnosis and monitoring of OSCC and OPMD [6]. Paxillin can be considered a reliable biomarker for identifying and comparing OPMDs and OSCC cancerous changes [7]. The JAK/STAT3 signaling pathway through IL-6 signaling causes cells to undergo EMT which results in the advancement of tumor spread and invasion. Studies show that higher IL-6 levels in OSCC tissues link to lymph node spread along with big tumors and severe histological characteristics [8]. Research studies have investigated how IL-6 functions in saliva to provide clinical values for both diagnosis and prognosis. The collection process of saliva offers easy accessibility together with minimal invasiveness and good patient cooperation primarily for high-risk subject groups who need repeated monitoring [9]. Salivary IL-6 has shown a strong ability to distinguish between OSCC patients and healthy individuals and people with potentially malignant conditions (based on data presented in [10]. Salivary interleukin-6 (IL-6) demonstrates strong potential as a non-invasive biomarker for the diagnosis and prognostication of oral squamous cell carcinoma, effectively distinguishing it from potentially malignant disorders and healthy individuals [11]. IL-6 stands as one of the primary biomarkers with the most stable level elevations between various research studies. The connection of IL-6 to tumor aggressiveness and disease stage progression makes it suitable for serving as a standalone biomarker to track malignant advancement. The potential therapeutic value of targeting IL-6 exists for oral oncology since anti-IL-6 agents are already being tested for cancer patients in other treatment settings [12]. Researchers face limitations when they use IL-6 marker despite its potential value for diagnostics. To achieve routine clinical application the challenges stem from various detection method inconsistencies combined with systemic inflammatory states and periodontal statuses and undefined reference specifications [13]. Clinical studies need to implement well-designed experiments with appropriate study sample sizes and controlled experimental factors to establish IL-6 as a valid biomarker in OSCC diagnosis. Therefore, it is of interest to evaluate interleukin-6 in oral squamous cell carcinoma as a salivary biomarker.

## Materials and Methods:

A total of 60 participants were enrolled in the study. These included 30 patients clinically and histopathologically diagnosed with Oral Squamous Cell Carcinoma (OSCC) and 30 age- and gender-matched healthy individuals who served as the control group. Informed consent was obtained from all participants.

## Inclusion criteria:

[1] Group I: Patients diagnosed with primary OSCC without prior treatment.

[2] Group II: Healthy individuals without any systemic illness or oral lesions.

## Exclusion criteria:

[1] Patients with a history of systemic inflammatory conditions.

[2] Individuals with previous history of radiotherapy, chemotherapy, or surgical intervention.

[3] Patients unwilling to provide consent.

## Saliva collection:

Unstimulated whole saliva was collected between 9:00 AM and 11:00 AM to minimize diurnal variations. Participants were instructed to refrain from eating, drinking, or oral hygiene procedures at least one hour before sample collection. Saliva was collected by the passive drool method into sterile tubes and immediately stored at -80°C until further analysis.

## Estimation of IL-6 levels:

Salivary IL-6 concentration was measured using a commercially available enzyme-linked immuno-sorbent assay (ELISA) kit following the manufacturer's protocol. All samples were processed in duplicates to ensure accuracy and consistency.

## Clinical staging:

OSCC patients were clinically staged using the TNM (Tumor-Node-Metastasis) classification as per the American Joint Committee on Cancer (AJCC) guidelines. Staging was recorded and correlated with the respective IL-6 levels.

## Statistical analysis:

Data were entered and analyzed using SPSS software version 26.0. Descriptive statistics such as mean and standard deviation were calculated. Intergroup comparisons were performed using the independent t-test, while ANOVA was used for evaluating differences across clinical stages. Pearson's correlation coefficient was used to assess the relationship between salivary IL-6 levels and clinical stage. A p-value of less than 0.05 was considered statistically significant.

## Results:

A total of 60 participants were included in the study, comprising 30 patients with Oral Squamous Cell Carcinoma (OSCC) and 30 healthy controls. The demographic distribution is presented in Table 1 (see PDF), showing a male predominance in both groups. The mean salivary IL-6 levels were significantly elevated in the OSCC group (112.4 ± 18.5 pg/mL) compared to the control group (22.8 ± 6.7 pg/mL), with a statistically significant difference (p< 0.001) (Table 2 - see PDF). Further analysis revealed a gradual increase in IL-6 levels with advancing clinical stage of OSCC. Patients in Stage I showed a mean IL-6 concentration of 78.3 ± 10.4 pg/mL, while Stage IV patients demonstrated the highest levels (139.2 ± 11.9 pg/mL) (Table 3 - see PDF). One-way ANOVA showed a statistically significant difference in IL-6 levels across all stages (p< 0.001).A strong positive correlation was observed between salivary IL-6 concentration and clinical stage (Pearson's r = 0.82, p< 0.001), indicating a direct association between IL-6 elevation and disease progression.

## Discussion:

Research investigated Interleukin-6 (IL-6) levels in oral fluid of patients with Oral Squamous Cell Carcinoma (OSCC) together with evaluation about how clinical staging affected these levels. Research results showed that patients diagnosed with Oral Squamous Cell Carcinoma presented higher IL-6 levels than people without cancer while the disease stage progression yielded greater IL-6 increases. This research shows that IL-6 presents itself as a promising non-invasive biomarker for detection and tracking of OSCC through saliva analysis. The tumor microenvironment benefits from IL-6 through its ability to boost angiogenesis processes which involves vascular endothelial growth factor (VEGF) upregulation and enhances both endothelial cell proliferation and migration [3]. Neovascularization happens to be essential for tumor growth and metastasis because it achieves nutrient and oxygen delivery to proliferating malignant cells. The relationship between OSCC aggressiveness and angiogenesis is strongly established so elevated IL-6 levels indicate such processes indirectly. IL-6 signaling activates oncogenes related to cell survival and apoptosis resistance through the JAK/STAT pathway [4]. IL-6 functions as a multifunctional cytokine which performs essential roles in immune regulation and inflammation and additionally takes part in oncogenesis [1]. The cancer-promoting effects of IL-6 occur through three main mechanisms which involve creation of new blood vessels and prevention of cell suicide along with increased tumor cell growth [2, 3]. Research investigating OSCC shows tissue, serum and saliva samples of affected patients have elevated IL-6 concentrations which indicate its contribution to tumor biology yet demonstrates its diagnostic potential [4, 5-6]. OSCC patients to possess elevated IL-6 levels in salivary samples than controls together with an association between increased levels and tissue stage progression [7]. Measurements of IL-6 levels showed increased values in OSCC patients which led them to suggest its potential as a disease activity indicator [8]. Laboratory data confirms previous results showing that IL-6 levels increase as clinical disease staging advances thus supporting the assessment of disease severity. The collection method for saliva as a diagnostic medium remains easy and cost-effective with non-invasive characteristics making it appropriate for mass screening programs within resource-limited areas [9]. Science demonstrates extensive use of saliva-based testing for systemic and local diseases such as oral cancers resulting in successful detection of IL-6, IL-8, TNF-α cytokines [10, 11]. Research shows that IL-6 plays a role in cancer microenvironment modification which helps tumors survive better and makes them more resistant to treatment agents [12]. The presence of high IL-6 levels in head and neck cancer patients leads to reduced survival rates and more frequent cancer recurrence according to research [13]. The vital biological functions of IL-6 support its status as both diagnostic tool and therapeutic objective.The study provides positive findings but still faces several research restrictions. Insufficient data points in this research as well as no ongoing assessments prevented the study from evaluating IL-6's future prognostic worth after treatment. Large study groups should exclude periodontal inflammation and systemic disease factors to properly interpret IL-6 levels [14, 15]. Future investigations must work towards combining IL-6 evaluation in saliva with other biomarkers to boost diagnostic effectiveness while examining its ability to forecast both treatment outcomes and survival patterns. The successful use of oral fluid biomarkers for clinical purposes requires proper establishment of universal reference intervals together with standardized test methods.

## Conclusion:

OSCC patients demonstrate higher IL-6 magnitude in saliva than healthy participants along with a relationship that grows proportionally to staging intensity. Thus, IL-6 levels in saliva can be used to develop an advantageous non-invasive tool for identifying and tracking OSCC disease activity and therapy evaluation.
